# Problem-based learning implementation in a health sciences blended-learning program in Argentina

**DOI:** 10.5116/ijme.5a7e.d85c

**Published:** 2018-02-23

**Authors:** María L. Cavicchia, Aana M. Cusumano, Daniela V. Bottino

**Affiliations:** 1 School of Medicine, Instituto Universitario CEMIC, Buenos Aires, Argentina

**Keywords:** Problem-based learning, blended learning, health sciences, Argentina

## Introduction

Problem-Based Learning (PBL) has been implemented as a teaching methodology in which contextualized problems are presented to students so that, by working in small groups, they can explore possible solutions. “PBL is an instructional (and curricular) learner-centered approach that empowers learners to conduct research, integrate theory and practice and apply knowledge and skills to develop a viable solution to a defined problem”.^1^ Barrows and Tamblyn^2^ defined PBL as a “very specific approach to education in medicine, supported by tools designed to facilitate a specific teaching-learning process”.

Wood^3^ explains that PBL has many advantages for the students because “it fosters active learning, improved understanding, and retention and development of lifelong learning skills.” Deep learning is nurtured through PBL as “students interact with learning materials, relate concepts to everyday activities, and improve their understanding.” However, from the tutors’ point of view, PBL can sometimes be frustrating and hard to manage since their role is not to teach but to facilitate knowledge and understanding. “Critical to the success of the approach is the selection of ill-structured problems… and a tutor who guides the learning process and conducts a thorough debriefing at the conclusion of the learning experience”.^1^

Based on the tenets of this approach, students get in groups to provide a solution to a given situation that represents a challenge to activate new knowledge. A tutor guides the students, and each group is asked to take responsibility for their learning, identifying what they already know, as well as when and where they should find the information they need to solve the problem.

There have been many experiences describing the implementation of PBL in different educational settings in the field of medicine.^4^^-^^6^ These experiences were first carried out in face-to-face, traditional classrooms. With the advent of technology, distance learning education – and blended-learning in particular - gained force and PBL started to be implemented in such modalities.

“Blended-learning, defined as the combination of traditional face-to-face learning and asynchronous or synchronous e-learning”^7^ which is put into practice using Virtual Learning Environments (VLE) such as online platforms or university campuses, brought about an entire set of new projects in many educational fields as well as in medicine. Programmes combining the strategies of PBL in blended-learning contexts were developed, and new ways of implementing PBL emerged with experiences which included online discussion forums,^8^ VLE,^9^^,^^10^ and simulation tools.^11^

These practices have been successful and have had positive results regarding training students in the Health Sciences.^7^^,^^8^^,^^10^ For example, Romero Gomez and Muñoz^10^ evaluated the impact of VLE instruction in the acquisition and construction of new knowledge in a Nursing School in Colombia. The researchers compared the performance of students using PBL techniques in face-to-face classrooms with the performance of students in VLE. Blended-learning modality students using PBL strategies have shown to perform better compared to those taking the same course in a traditional, face-to-face classroom.

Overall, PBL and blended-learning have grown as a promising scheme for the implementation of other projects in the training of Health Sciences students. The case that follows is an example of a course designed for the School of Medicine of a Health Science University Center in Argentina. Thus, the purpose of this article is to describe the experience of implementing such course combining PBL and blended-learning.

### Context of implementation

The institution where this experience was carried out is in the northern area of the city of Buenos Aires. The educational center is privately-run and has provided instruction and training in the field of Health Sciences since 1998. It offers graduate and post-graduate programmes in areas such as medicine, nursing, and physical therapy, among others. The number of students in the School of Medicine is not a large one, reaching up to around 40 students per course.

Courses are delivered in face-to-face and blended-learning modality. For the blended-learning courses, students attend regular lessons at the center and access an online learning platform which allows them to download materials, participate in forums, solve online activities, and interact with their teachers.

A four-week PBL non-mandatory course is offered every year, and it is led by tutors who are specialists in medicine as well as experienced teachers in the field of PBL.  Although the PBL course is non-mandatory, it is a requirement for all the students to participate in the course at some point in the programme to be allowed to take other subjects.

### The experience

The PBL-blended-learning course this article reports was designed for the students in the School of Medicine. These students are systematically trained in the integration of activities that foster the development of skills and competencies in their field of study. However, it has been observed that there was a need to enhance students’ technological skills in terms of using and participating in the online learning platform. This is where the plan of combining PBL and blended-learning came into action.  Accordingly, the main objective of the course was to help students improve their performance using the learning platform through the application of PBL strategies.

The experience took place in 2013 and the course comprised face-to-face meetings and online interactions on the learning platform. Even though the regular PBL course is offered to all the students in the School of Medicine, for this project, only students in 4th and 5th years were invited to participate. The choice of senior students responded to the need to have experienced participants who had already taken face-to-face PBL courses for at least three years and who could show some level of expertise in the application of PBL strategies. A total of 68 students (36 in 4th year and 32 in 5th year) took part in this version of the non-mandatory, blended-modality of PBL.

The students met their tutors twice a week for face-to-face lessons and then these meetings were complemented with online tasks on the platform. The selected tutors were requested to be highly committed to the tasks that needed their attention on the learning platform as well as to have plenty of availability for students to contact them, especially in face-to-face interactions which occurred twice a week.

For the face-to-face meetings, the tutors in charge of the course prepared vignettes on health issues which had a strong contextualized, social component representing main health problems in our country. The students were presented with these PBL cases and, after discussing possible solutions on the platform, they came back to the face-to-face meetings to check doubts or share discoveries.

The activities on the learning platform were mainly based on discussion forums for the students to intervene in groups. Two tutors oversaw the interactions in the forums, which lasted for two weeks, combined with two other weeks of face-to-face lessons.

Evaluation of the students was systematically carried out using rubrics, which the students had access to as soon as they started the course. The purpose of sharing the rubrics with the students was based on the premise that they had to be familiarized with the evaluation tool before starting their participation; this would raise the students’ awareness of the performance items that were to be observed for their evaluation. These rubrics were designed to evaluate the main strategies that applying PBL required, such as group work, searching for information and the quality of the information searched for, generating hypotheses, and developing critical thinking skills.  Each tutor completed the rubrics with information about the students’ participation during the face-to-face meetings and the online interactions. The rubrics were also used as guidelines for the feedback the students received in one-to-one and group feedback sessions. Besides, the students were requested to complete an online survey to evaluate their tutors and self-evaluate their performance. This survey was implemented to get to know the students’ opinion about the PBL activity and their perceptions regarding the course. Additionally, some questions delved into how the students felt with their tutors’ guidance and whether they believed a certain skill had been developed as a result of their participation in the course.

### Issues and challenges

Even though the institution has recognized that innovation in education is embodied in the integration of blended-learning with all the courses that are offered every year, it has been perceived that culturally speaking teachers and students were, at the time of implementation, still learning how to incorporate the online learning platform to their everyday teaching-learning practices. In this context, the implementation of the PBL-blended-learning course we have reported here was not an easy task. This meant sorting out some issues that were observed throughout the course delivery.

The use of the online learning platform is an issue that needs further reinforcement. The students reported that they found difficulties to use the platform since they did not have previous experience and knowledge in technological skills, such as accessing the platform or publishing their posts in the forums. Thus, an increase in the use of the platform and the students’ participation in VLE is still a challenge to consider.

Regarding the students’ participation either in face-to-face meetings or online communications, a point that deserves attention is the fact that those students who showed a greater ability to express their ideas orally, also outperformed in the written interactions on the platform. This was also a challenge for the tutors because they had to find strategies so that those students who tended to participate less were able to express what they thought.

Particularly interesting is the fact that most written interactions on the learning platform were published by the students who were less participative in oral, face-to-face activities. Additionally, the students who outperformed both in written and oral interactions were able to show excelling summarizing abilities which resulted in more appropriate acknowledgment of sources and better understanding of the contents. We also noticed that some students committed plagiarism when posting their publication in the forums. This was yet another challenge for both the tutors, who had to deal with raising awareness of avoiding such practices and their peers, who noticed this and expressed their discontent.

Regarding PBL strategies applied on the platform and skills development, most of the students showed improvements in areas such as searching for information, reflecting upon presented cases before intervention, debating with peers, regularly attending lessons, and communicating with peers and tutors.

As for the tutors’ side, the reduced number of tutors was also a challenge to take on. Since there were only two tutors, they were overburdened and had lots of activities to supervise.

### Lessons learned

The experience shared in this article provided us with a unique opportunity to consider the online learning platform as an invaluable tool to implement PBL strategies. In the light of the analysis of the issues and challenges, the initial objective we had set was met, with the caveat that the students at the institution still need to work on their technical skills development and accept the incorporation of the learning platform as part of their everyday learning.

Despite this, the students and the tutors participating in this project positively welcomed every task that they were requested to complete and every challenge that they were faced with. Both the students and the tutors resorted to PBL strategies to resolve the issues described above; this provided them with an opportunity to further develop PBL skills in VLE.

Areas to consider for further implementations include the number of tutors per group of students and the training of students in the use of the learning platform early in the programme. Furthermore, tutoring students in VLE requires a whole new set of skills that the tutors had to learn as they were taking part of the experience. Although the pedagogical, face-to-face strategies that PBL tutors bring with them to the VLE were useful, they had to put into practice other abilities to optimize their interventions and guidance.

## Conclusions

Implementing a PBL-blended-learning course as the one we have described is a viable alternative which sets the grounds for innovative practices in the training of medical students. This course allowed us to provide students with the opportunity to develop abilities in two fields: technology and PBL.

In our view, PBL tutors willing to participate in blended-learning projects such as this one also need proper training to be able to maximize their abilities and strategies. This experience has allowed tutors to broaden their experience in PBL and blended-learning further.

Regarding the students’ opinion about this course, the majority agreed that this blended-learning experience allowed them to envisage their prospectus professional practices.

Since the implementation of this course, new projects, which include the incorporation of Information and Communication Technologies, have been carried out and this has motivated faculty staff to explore other ways of teaching and learning in the School of Medicine. We consider that it is highly necessary to continue monitoring such experiences to gain more expertise in the field of PBL and blended-learning.

### Conflicts of Interest

The authors declare that they have no conflict of interest.
